# Gestational diabetes and pregnancy outcomes - a systematic review of the World Health Organization (WHO) and the International Association of Diabetes in Pregnancy Study Groups (IADPSG) diagnostic criteria

**DOI:** 10.1186/1471-2393-12-23

**Published:** 2012-03-31

**Authors:** Eliana M Wendland, Maria Regina Torloni, Maicon Falavigna, Janet Trujillo, Maria Alice Dode, Maria Amélia Campos, Bruce B Duncan, Maria Inês Schmidt

**Affiliations:** 1 Federal University of Health Sciences, Porto Alegre, Brazil; 2 São Paulo Federal University, São Paulo, Brazil; 3 Federal University of Rio Grande do Sul, Porto Alegre, Brazil; 4 Federal University of Pelotas, Pelotas, Brazil; 5 Conceição Hospital, Porto Alegre, Brazil

## Abstract

**Background:**

Two criteria based on a 2 h 75 g OGTT are being used for the diagnosis of gestational diabetes (GDM), those recommended over the years by the World Health Organization (WHO), and those recently recommended by the International Association for Diabetes in Pregnancy Study Group (IADPSG), the latter generated in the HAPO study and based on pregnancy outcomes. Our aim is to systematically review the evidence for the associations between GDM (according to these criteria) and adverse outcomes.

**Methods:**

We searched relevant studies in MEDLINE, EMBASE, LILACS, the Cochrane Library, CINHAL, WHO-Afro library, IMSEAR, EMCAT, IMEMR and WPRIM. We included cohort studies permitting the evaluation of GDM diagnosed by WHO and or IADPSG criteria against adverse maternal and perinatal outcomes in untreated women. Only studies with universal application of a 75 g OGTT were included. Relative risks (RRs) and their 95% confidence intervals (CI) were obtained for each study. We combined study results using a random-effects model. Inconsistency across studies was defined by an inconsistency index (I^2^) > 50%.

**Results:**

Data were extracted from eight studies, totaling 44,829 women. Greater risk of adverse outcomes was observed for both diagnostic criteria. When using the WHO criteria, consistent associations were seen for macrosomia (RR = 1.81; 95%CI 1.47-2.22; p < 0.001); large for gestational age (RR = 1.53; 95%CI 1.39-1.69; p < 0.001); perinatal mortality (RR = 1.55; 95% CI 0.88-2.73; p = 0.13); preeclampsia (RR = 1.69; 95%CI 1.31-2.18; p < 0.001); and cesarean delivery (RR = 1.37;95%CI 1.24-1.51; p < 0.001). Less data were available for the IADPSG criteria, and associations were inconsistent across studies (I^2 ^≥ 73%). Magnitudes of RRs and their 95%CIs were 1.73 (1.28-2.35; p = 0.001) for large for gestational age; 1.71 (1.38-2.13; p < 0.001) for preeclampsia; and 1.23 (1.01-1.51; p = 0.04) for cesarean delivery. Excluding either the HAPO or the EBDG studies minimally altered these associations, but the RRs seen for the IADPSG criteria were reduced after excluding HAPO.

**Conclusions:**

The WHO and the IADPSG criteria for GDM identified women at a small increased risk for adverse pregnancy outcomes. Associations were of similar magnitude for both criteria. However, high inconsistency was seen for those with the IADPSG criteria. Full evaluation of the latter in settings other than HAPO requires additional studies.

## Background

The definition of gestational diabetes mellitus (GDM) as any degree of glucose intolerance with onset or first recognition during pregnancy is largely accepted. However, the precise level of glucose intolerance characterizing gestational diabetes has been controversial over the last three decades.

In 1979-1980, U.S. National Diabetes Data Group (NDDG) [1] and the World Health Organization (WHO) [2] established that the 2 h 75 g oral glucose tolerance test (OGTT) should be the main diagnostic test for glucose intolerance outside of pregnancy.

Regarding glucose intolerance during pregnancy, two different approaches were taken. The NDDG opted, in pregnancy, to maintain the 3 h 100 g OGTT test, largely used and evaluated in the USA. The American Diabetes Association (ADA) and many other medical associations around the world adopted over the years this 3 h 100 g OGTT test. In so doing, different cutoffs for the diagnosis of GDM were chosen, one of the issues being the difficulty in converting blood glucose values from the original studies done in the 1960s and 1970s [1,3-5] to their plasma equivalents analyzed using new analytic methods.

The WHO adopted the 2 h 75 g OGTT in pregnancy, recommending the same diagnostic cut points established for the diagnosis of impaired glucose tolerance outside of pregnancy [2,3]. In 1999, WHO clarified that GDM encompassed impaired glucose tolerance and diabetes (fasting ≥ 7 mmol/l or ≥ 126 mg/dl; 2 h plasma glucose ≥ 7.8 mmol/l or 140 mg/dl) [6] and, over the years has maintained their recommendations.

More recently, the International Association of the Diabetes in Pregnancy Study Group (IADPSG), after extensive analyses of the Hyperglycemia and Adverse Pregnancy Outcomes (HAPO) study [7], recommended new diagnostic criteria for GDM [8] based on the 2 h 75 g OGTT: a fasting glucose ≥ 5.1 mmol/L (92 mg/dl), or a one hour result of ≥ 10.0 mmol/L (180 mg/dl), or a two hour result of ≥ 8.5 mmol/L (153 mg/dl).

A considerable number of prospective studies have now investigated the use of a 2 h 75 g OGTT in pregnancy in relation with various pregnancy outcomes, thus allowing evaluation of these two main diagnostic criteria. Thus, the purpose of this study is to summarize, through a systematic review, the association of GDM, as diagnosed by the WHO and the IADPSG criteria, with adverse pregnancy outcomes, in untreated women. In so doing, the applicability of the IADPSG criteria to non-HAPO settings is also evaluated.

## Methods

### Criteria for considering studies for this review

#### Types of study

Cohort studies (prospective or retrospective) were considered for inclusion in this systematic review if they provided sufficient information to estimate the associations of the WHO and/or the IADPSG criteria with related perinatal and maternal outcomes.

To avoid selection bias, we included only studies that applied the OGTT universally to all participants. We therefore excluded studies applying the OGTT only in women with certain clinical risk factors (such as family history, obesity, previous GDM) or in those positive in pre-OGTT glucose screening (with, for example, a 50 g challenge test and/or a fasting glucose). We also excluded studies that did not distinguish pre-gestational diabetes mellitus from GDM, those not allowing the distinction between treated and untreated groups, and those not reporting outcomes for women classified as having a normal OGTT.

#### Types of participants

We accepted studies which included women of any race, parity, age, body weight or other socio-demographic characteristics.

#### Types of diagnostic tests

Only studies based on a 2 hour 75 g OGTT performed during the 2^nd ^or the 3^rd ^trimesters were included, and only if they provided results for a diagnosis based on at least the 2 h post-load glucose. Studies based on capillary glucose measurements were included.

#### Types of outcome measures

We decided to analyze, as perinatal outcomes, large for gestational age births and macrosomia (as defined by the authors), as well as perinatal mortality (fetal death and early neonatal death). Regarding maternal outcomes, we chose to analyze cesarean delivery and preeclampsia according to individual study definitions.

### Search methods for the identification of studies

The search strategy used the following general terms, adapted to each database: "gestational diabetes" or "glucoseintolerance" and the appropriate terms for each of the maternal and perinatal adverse outcomes specified above. Specific terms used for the electronic search are detailed in the Additional file 1: Description of the electronic search strategy used to perform the literature search.

We searched ten electronic databases (MEDLINE, EMBASE, LILACS, the Cochrane Library (CENTRAL), CINHAL, WHO-Afro library, IMSEAR, EMCAT, IMEMR and WPRIM) for articles published from inception up to March 15, 2011. No language or country restrictions were applied. We also searched for additional studies from classical review articles. The reference lists of all articles selected for full text reading were reviewed for additional potentially eligible studies.

### Data collection and analysis

#### Selection of studies

All citations identified were entered into an electronic database and duplicates were deleted. Initially, two investigators independently screened the titles and abstracts of potentially relevant studies for eligibility. When the information was not sufficient to determine if the article was eligible for inclusion, the article's full text was obtained for further evaluation. Discrepancies were discussed until consensus was reached.

#### Data extraction and management

Two independent investigators reviewed the eligible studies and extracted data using a standardized form prepared for this review. Disagreements were discussed and resolved in a consensus meeting. When raw quantitative data were not reported, approximate values were obtained from the figures or calculated from percentages.

#### Assessment of methodological quality

The methodological quality of the included studies was assessed by examining factors which might affect the strength of the association between glucose levels and outcomes. In particular, the following factors were assessed in each study: i) adequate selection of participants: consecutive recruitment from prenatal clinics; ii) adequate standardization of the glucose tolerance test (pre-analytic factors such as anhydrous glucose, plasma immediately separated or kept with glycolytic inhibitors and kept refrigerated until centrifugation; and analytic factors such as enzymatic method of measurement and laboratory quality control); iii) adequate report of losses to follow up and; iv) medical staff blinded to OGTT results.

#### Data synthesis

Data for the WHO and the IADPSG criteria were aggregated and presented as relative risk (RR) with 95% confidence interval (CI). Meta-analysis data were combined using random-effect models, with restricted maximum-likelihood (REML) estimation. The statistical analysis was performed using the R version 2.11.1 software, package metafor version 1.6-0 [9]. As our aim was to investigate diagnostic criteria based on their capacity to predict GDM-related outcomes for classification purposes rather than for etiological investigation, all statistical analyses were crude, without adjustment for potential confounders.

#### Assessment of heterogeneity

Overall results were calculated based on the random effects model. We assessed heterogeneity using the Cochrane's χ2 statistics with a significance level of 0.10. Inconsistency indexes (I^2^) were also calculated, and a value greater than 50% was considered an indicator of high inconsistency between studies [10].

#### Sensitivity analysis and assessment of publication bias

We did sensitivity analyses in order to examine the influence of the HAPO study and Brazilian Study of Gestational Diabetes (EBDG) on the magnitude and consistency of associations with outcomes. In addition to REML, we also aggregated data with other variance estimators (Maximum Likelihood, Empirical Bayes, Sidik-Jonkman and DerSimonean and Laird) and with a fixed effect model in order to assess the robustness of the model.

Publication bias was tested using a funnel plot and Egger's test based on weighted regression [11].

The full database for the EBDG study was available which allowed analysis for both criteria for all outcomes. The EBDG study was approved by local institutional review boards and informed consent was obtained from all participants. Data from the other studies were obtained from published articles cited in the list of references.

## Results

### Results of the literature search

**Figure 1:**
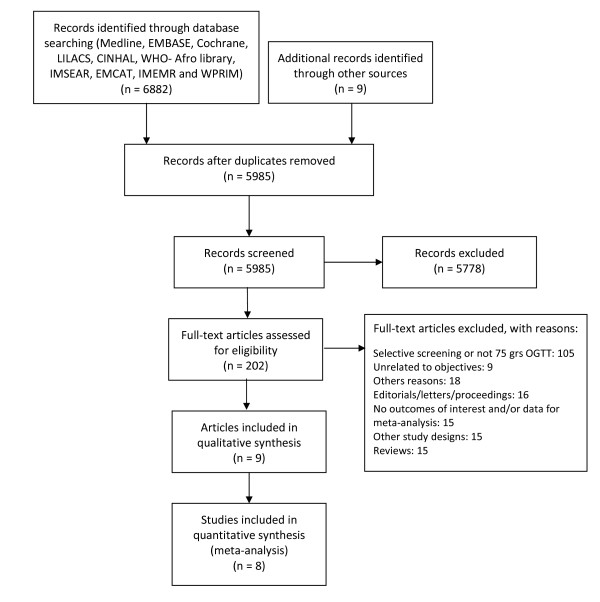
**Flow chart for the process of study identification and inclusion**.

Figure 1 (flow chart) describes the process of study identification and inclusion, and the reasons for exclusions. Our search identified 5985 references, without duplicates. Nine citations were retrieved from the reference lists of the full-text articles. After revising all titles and abstracts, 202 potentially relevant citations were identified and full papers were obtained for all. A total of 9 publications pertaining to 8 studies met the selection criteria and were included in this systematic review. For a description of excluded studies, see Additional file 2: List of excluded articles. The full database for the EBDG study was available which allowed analysis for both criteria for all outcomes.

### Included studies

Data on the adverse outcomes associated with diagnostic criteria were extracted from 8 studies (9 publications) [7,12-19], all of which were published in full in peer reviewed journals (Table 1). Of the 8 included studies, three were retrospective and information was gathered through data linkage or chart review [15,17,18]. One study was performed in the United States [13], one in Asia [18], two in the Middle East [17], one in Europe [12], two in Latin America [14,16] and one was a multi country study [7,19]. All but one study [12] used venous plasma glucose based on the oral glucose tolerance test to diagnose GDM. Taken together, the 8 studies provided information on a total of 44829 women. The database of the Brazilian Study of Gestational Diabetes (EBDG) [16] was used to generate data when results from other studies were not available from the published literature.

**Table 1:** Main characteristics of the studies included

Study	N	Incidence of GDM (%)^1 ^	Ethnicity	Maternal age ^2 ^	Gestational age at OGGT (weeks) ^2 ^		Pre-gravid BMI^2 ^	Study criteria for GDM treatment ^3 ^mmol/L
Aberg 2001 [12] Sweden	4773	5.2	Not reported	Not reported	25-30		Not reported	2 hs PG > 9.0

Black 2010 [13] USA	8711	19.4	White: 7.2; Black: 10.;1 Hispanic: 74.4; Asian: 7.4; Other: 0.9	29.1 ± 5.9	26.7 ± 2.9		27.5 ± 6.1	At least 2 abnormal values: FPG ≥ 5.5; 1 hPG ≥ 10.8; 2 hPG ≥ 8.9

EBDG 2001 [16] Brazil	4998	7.5	White: 44.9 Mixed: 41.1 Black: 13.6 Other: 0.4	27.8 ± 5.5	24-28		23.4 ± 4.0	2 hPG ≥ 10.0

Forsbach 1997 [14] Mexico	667	16.0	Hispanic	18 - 44	34.2 (24-40)		Not reported	2 hPG > 10.0

HAPO 2008, 2010 [7,19] multi-countries	23316	11.4	White: 48.3 Black: 11.6 Hispanic: 8.5 Asian: 29.0 Other: 2.6	29.2 ± 5.8	27.8 ± 1.8	27.7 ± 5.1	FPG > 5.8; or 2 hPG > 10.0; or RPG ≥ 8.9	

Khan 1994 [15] Pakistan	1278	4.9	Not reported	26.7 ± 4.6	16 - 20	Not reported	. At least 2 abnormal values: FPG > 5.8; 1 hPG > 10.3; 2 hPG > 7.8/3 hPG > 6.8	

Shirazian 2008 [17] Iran	670	12.1	Not reported	NR	24-28	Not reported	At least 2 abnormal values: FPG ≥ 5.5; 1 hPG ≥ 10.0; 2 hPG ≥ 8.3	

Sugaya 2000 [18] Japan	416	32.5	Asian	30.3 ± 4.3	25.4 ± 8.2	22.4 ± 3.8	At least 2 abnormal values: FPG ≥ 5.5; 1 hPG ≥ 10.0; 2 hPG ≥ 8.3	

GDM: Gestational diabetes; OGTT: Oral glucose tolerance test; PG: Plasma glucose; FPG: Fasting PG; RPG: random PG

^1 ^GDM incidence according to WHO except for Black et al., who used the IASDPG criteria

^2 ^Range or mean ± SD

^3 ^The meta-analyses only included untreated women, as defined in each study

We only analyzed results for untreated women. Because of the ethical need to offer treatment to women identified as having GDM by the diagnostic criteria in use at the moment of the testing, some studies excluded such women and others presented results permitting the separation of those who received treatment. As seen in Table 1 in some cases, this resulted in study samples with a very narrow glucose range.

Quality assessment of the studies included is summarized in Table 2. Most of the studies (6/8) had adequate selection of participants, half of them presented adequate test standardization and reported losses to follow-up and only one study informed that medical staff was blinded to OGTT results.

**Table 2:** Assessment of methodological quality of included studies

Study	Adequate selection of participants	Adequate test standardization	Adequate report of losses to follow-up	Medical staff blinded to OGTT results
Aberg, 2001 [1]	Uncertain	No	Uncertain	No
Black, 2010 [13]	Yes	Uncertain	Yes	No
EBDG, 2001 [16]	Yes	Yes	Yes	No
Forsbach, 1997 [14]	Yes	Yes	Uncertain	No
HAPO, 2008, 2010 [7,19]	Yes	Yes	Yes	Yes
Khan, 1994 [15]	Yes	Uncertain	Uncertain	Uncertain
Shirazian, 2008 [17]	Yes	Yes	No	Uncertain
Sugaya, 2000 [18]	Uncertain	Uncertain	Yes	No

### Perinatal outcomes

**Figure 2:**
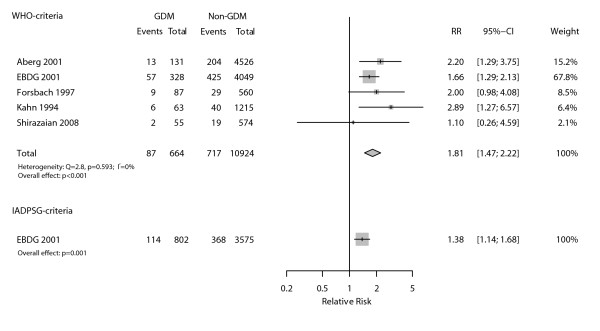
**Relative incidence (RR) of macrosomia among those with and without gestational diabetes as defined by WHO and IADPSG diagnostic criteria**.

As seen in Figure 2, a total of five studies allowed evaluation of the association between GDM diagnosed through WHO criteria and fetal macrosomia (defined by authors as birth weight > 4000 g, except Aberg et al [12]). The corresponding pooled relative risk (RR) was 1.81 (95%CI 1.47-2.22; p < 0.001), with very homogenous results across studies (I^2 ^= 0%). We did not identify any published study allowing evaluation of macrosomia according to the IADSPG diagnostic criteria. Therefore, we performed this analysis using the EBDG database and the RR was 1.38 (95%CI 1.14-1.68; p = 0.001).

**Figure 3:**
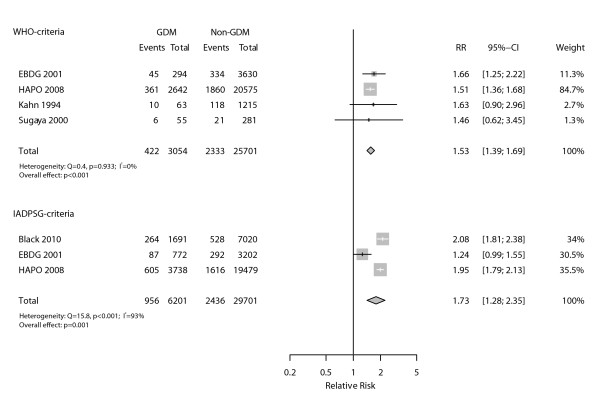
**Relative incidence (RR) of large for gestational age infants among those with and without gestational diabetes as defined by WHO and IADPSG diagnostic criteria**.

When we assessed large for gestational age births, defined as birthweight ≥ 90^th ^percentile for gestational age (Figure 3), the association seen for the WHO criteria (four studies) was slightly lower (RR = 1.53, 95% CI 1.39-1.69; p < 0.001), and very homogeneous across studies (I^2 ^= 0%). Regarding the IADPSG criteria, the large inconsistency across the three studies evaluated (I^2 ^= 93%) limited the validity of the pooled RR (1.73; 95%CI 1.28-2.35; p = 0.001).

**Figure 4:**
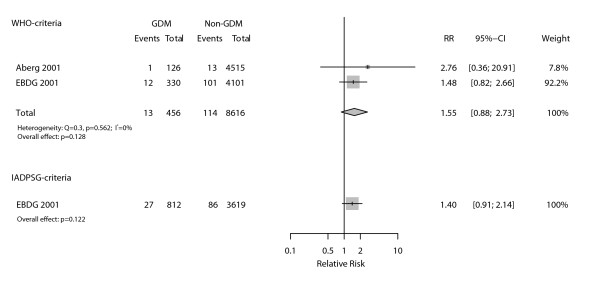
**Association between perinatal mortality and gestational diabetes as defined by WHO and IADPSG diagnostic criteria**.

With regard to perinatal mortality (Figure 4), only two studies provided sufficient data for the evaluation of the WHO criteria. Their summary produced a homogenous (I^2 ^= 0%) association of clinically relevant size but lacking statistical significance (RR = 1.55, 95%CI 0.88-2.73; p = 0.128). No studies were available to evaluate the IADSPG diagnostic criteria with regard to perinatal mortality. Analysis with the EBDG database yielded similar, non-statistically significant, results (RR = 1.40, 95%CI 0.91-2.14; p = 0.122).

### Maternal outcomes

**Figure 5:**
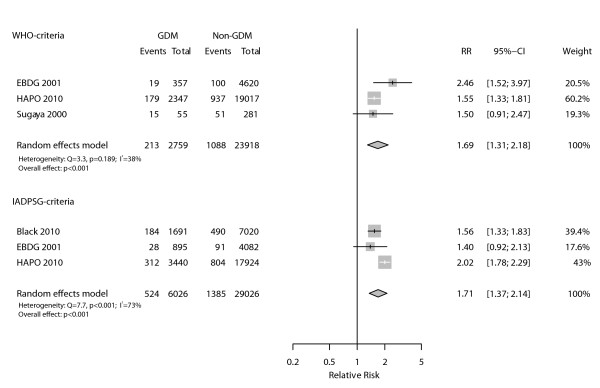
**Association between preeclampsia and gestational diabetes as defined by WHO and IADPSG diagnostic criteria**.

Four studies [13,16,18,19] provided data on preeclampsia, one of which included cases of transient hypertension or unspecified hypertension in the same group as preeclampsia [13]. As presented in Figure 5, there was a positive and statistically significant association between the WHO diagnostic criteria and preeclampsia (pooled RR = 1.69, 95%CI 1.31-2.18; p < 0.001) with reasonable consistency across the three studies evaluated (I^2 ^= 38%). When analyzed by the IADPSG criteria, the pooled RR for the same outcome was of similar magnitude (RR = 1.71, 95%CI 1.38-2.13; p < 0.001), but aggregated very inconsistent results for the three available studies (I^2 ^= 73%).

**Figure 6:**
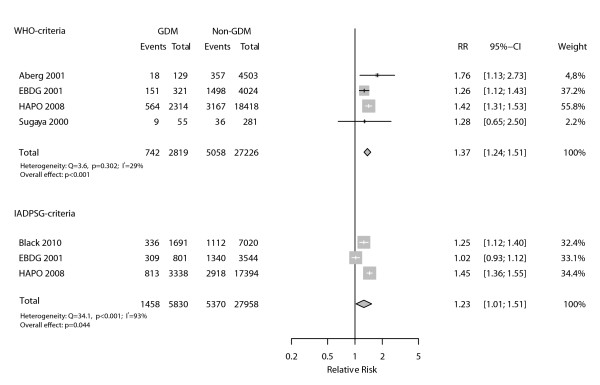
**Association between cesarean delivery and gestational diabetes as defined by WHO and IADPSG diagnostic criteria**.

Figure 6 presents data pertaining to studies with sufficient information to evaluate diagnostic criteria as predictors for cesarean delivery. Both diagnostic criteria detected women with an increased risk, the association being slightly higher when GDM was diagnosed according with the WHO criteria (RR = 1.37, 95%CI 1.24-1.51; p < 0.001) than with the IADSPG criteria (RR = 1.23, 95%CI 1.01-1.51; p = 0.044). The associations were consistent across the four studies analyzed according to the WHO criteria (I^2 ^= 29%), but were inconsistent across the three studies that used the IADSPG criteria (I^2 ^= 93%).

### Sensitivity analyses and assessment of publication bias

**Figure 7:**
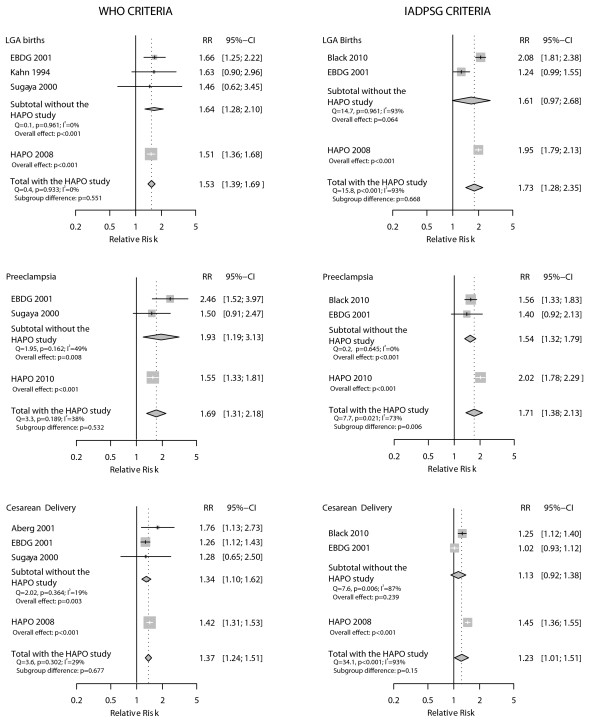
**Sensitivity analysis excluding the HAPO study**.

Because the HAPO study was used to generate the IADPSG criteria, we performed post hoc subgroup analysis excluding the HAPO study (Figure 7) for all outcomes for which it contributed data. For the analyses of the IADPSG criteria, the pooled RRs after exclusion were always somewhat smaller than the RR for the HAPO study alone. After exclusion, the pooled RRs remained statistically significant for preeclampsia (p = 0.006), but not for large for gestational age and cesarean delivery. For the analyses of the WHO criteria, the pooled RRs excluding HAPO were generally greater than the RR for the HAPO study alone, although not statistically significantly so; and remained statistically significant after the exclusion.

**Figure 8:**
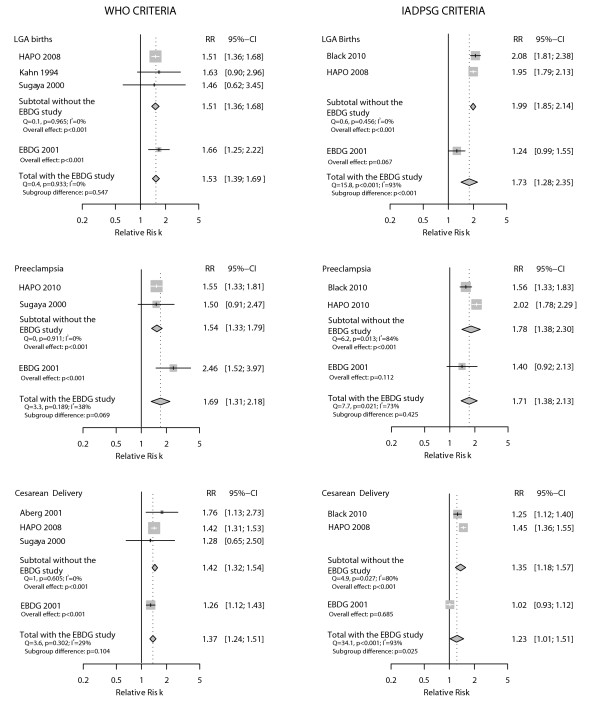
**Sensitivity analysis excluding the EBDG study**.

As the EBDG study was used in all analysis, sometimes using individual patient data from the original database, we also performed post hoc sensitivity analyses excluding this study (Figure 8). For the analyses of the IADPSG criteria, this exclusion led to somewhat increased pooled RRs, statistically different from the RRs for the EBDG study for large for gestational age and cesarean delivery; after the exclusion of the EBDG study, the pooled RRs remained statistically significant. For the analyses of the WHO criteria, pooled RRs were generally smaller after exclusion of the EBDG study, but remained statistically significant.

It was not possible to assess the influence of the HAPO study on macrosomia, as no HAPO data were available for this outcome. With respect to the influence of the EBDG study, its exclusion led to an increase in the pooled RR for the WHO criteria (from RR 1.81 to RR 2.17).

Meta-analyses performed with different variance estimators had little impact on the RR and on the I^2^. Stronger associations were found with fixed-effect models for outcomes assessed with the IADPSG criteria (Additional file 3: Meta-analyses performed with different variance estimators to generate pooled relative risks for the IADSPG and WHO criteria for gestational diabetes in the prediction of pregnancy outcomes).

Funnel plots and the Egger's regression asymmetry test (data not shown) did not reveal evidence for publication bias, although small number of studies compromised this analysis.

## Discussion

This is the first systematic review to assess the magnitude of the associations between different GDM diagnostic criteria and several clinically relevant outcomes. We focused analyses on the two main diagnostic criteria currently under debate for a 75 g OGTT - i.e., those recommended by the WHO and those recently proposed by the IADSPG on the basis of pregnancy outcomes. In addition to providing estimates for the magnitude of the increased risk predicted by these two criteria, we also evaluated the application of the IADPSG criteria to settings other than that of the HAPO study.

Our summary estimates of relative risk demonstrate that GDM diagnostic criteria based on both the WHO and the IADPSG criteria predict perinatal and maternal adverse outcomes. The strength of the crude associations found ranged from 1.23 (95% CI 1.01-1.51) for cesarean delivery, to 1.81 (95% CI 1.47-2.22) for macrosomia. For the three outcomes for which meta-analyses were possible for both criteria (large for gestational age, preeclampsia and cesarean delivery), the magnitude of the effects were similar for the WHO and the IADPSG criteria (1.53 vs. 1.73; 1.69 vs. 1.71; 1.37 vs. 1.23, respectively), although the inconsistency across studies limited aggregate estimation for the IADPSG criteria. Sensitivity analyses excluding either the HAPO or the EBDG study did not materially change the magnitude of these associations (changes varying between 1 and 13%).

It is important to note that these crude associations are very small within a diagnostic context. Two reasons may explain the small associations found. First, both GDM criteria, especially the IADPSG criteria, identify lesser degrees of hyperglycemia when compared to other ones, such as those previously recommended by the ADA [20]. Second, as all the studies analyzed in this review excluded women receiving specific treatments for GDM (see Table 1), the range of hyperglycemia classified as GDM represents a mild degree of hyperglycemia. Given the continuum of risk in the association between plasma glucose and pregnancy outcomes [7], if both criteria were applied to a broader spectrum, such as the one seen in the usual clinical setting, which includes women at greater risk given their higher glucose level, the association should be stronger. Nevertheless, even if GDM diagnostic criteria were to reach relative risks close to 3 for these adverse outcomes in such settings, the relative risks would still be unlikely to reflect major diagnostic discrimination in terms of post test probabilities [21]. This fact suggests the importance of investigating the contribution to risk discrimination of other factors, besides glycaemia, for these outcomes.

It is also important to interpret the heterogeneity found across studies, most seen for the IADPSG criteria. Potential reasons for heterogeneity include different population characteristics, study design and nature of the diagnostic criteria. As sensitivity analyses examining the influence of the EBDG and the HAPO studies did reveal some changes in the heterogeneity found, particularities about each of these study settings need to be considered. The HAPO study is a large multi-country study conducted from 2000 to 2006 with a strict research protocol. The EBDG study is a multicenter study conducted in Brazil in the 1990's with a less strict protocol, in a scenario of less intervention, following women with a wider range of hyperglycemia. A more strict protocol, with more control over incomplete fasting, such as that seen in the HAPO study, could produce larger associations with the IADPSG criteria, which diagnoses an appreciable fraction of cases on the basis of the fasting value. In fact, the application of the IADPSG criteria in two published studies [13,22] and in the EBDG database showed that the fasting value identified over 70% of all cases of GDM so defined, while when these criteria were applied to the HAPO study as a whole, the fasting value identified only about 50% of cases. However, as this rate in HAPO varied from 24% (Thailand) to 74% (Barbados)[23], whether these differences resulted from incomplete fasting or from other specific study or population particularity cannot be concluded from current information. The lack of blinding to glucose levels in most studies (except HAPO) could lead to GDM treatment, and thus reduce the magnitude of the associations; so we excluded such women. Although undetected intervention may still be present even after these exclusions, for example, diet, it is unlikely that this would cause more heterogeneity in the IADPSG than in the WHO analyses.

One hypothesis is that the IADPSG criteria are more vulnerable to heterogeneity across different settings because they allow that diagnosis be made on the basis of only one out of three possible measures (fasting, 1 h and 2 h). Given population variability in terms of the probability of being positive by fasting and post load values, as well as in terms of the possibility of having incomplete fasting (drank coffee or tea with sugar; for example), more heterogeneity could be found for the IADPSG criteria. Another possibility, worth exploring in future studies, is whether the heterogeneity stems from differences in the prevalence or characteristics of obesity in the underlying populations.

Additionally, since the IADPSG criteria were derived from the HAPO study, lower performance of these criteria in non-HAPO settings is to be expected. For large for gestational age and for cesarean delivery, results remained inconsistent across studies after excluding HAPO, which makes questionable the estimates of pooled RRs generated for these outcomes (the pooled RRs found were lower and not statistically significant). For preeclampsia, results across studies became consistent, but with an RR (1.54; 95% CI 1.32-1.79) smaller (p = 0.006) than that found for the HAPO study (2.02; 95%CI 1.78-2.29).

Our study has some limitations. First, few studies were available to evaluate important outcomes such as perinatal mortality and long-term outcomes in offspring. Yet, positive associations were found for macrosomia and pregnancy related hypertension, two clinically relevant outcomes. Second, as we excluded studies conducted with selective screening and studies not allowing analysis of untreated women, we eliminated several otherwise good studies which were included in other reviews on GDM screening [24]. Publication bias could not be excluded because of the small number of studies examined.

Our study also has several strong points, including its originality, extensive search strategy, inclusion of studies independent of language, strict methodological rigor, assessment of study quality, and sensitivity and subgroup analyses to investigate the applicability of the IADPSG criteria in settings other than the HAPO study.

## Conclusions

In conclusion, the meta-analyses of studies examining the WHO and IADPSG criteria demonstrate small increased risk for adverse pregnancy outcomes, with generally similar magnitudes of associations for each criteria. For the WHO criteria, positive associations were consistent across studies. For the IADPSG criteria, additional studies will be needed to adequately estimate the magnitude of associations when applied to non-HAPO settings.

## Competing interests

All authors have completed the Unified Competing Interest form, declaring the absence of financial interests that may be relevant to the submitted work.

## Authors' contributions

MIS participated in all the aspects of the project and was the overall supervisor. Additional participation was as follows: Writing the protocol: EMW and MRT; developing the search strategy: EMW; searching and selecting trials: EMW, MRT, MAC, MAD, JT; data extraction: JT, EMW, MRT; data analysis: MF; drafting and final review: All. All authors read and approved the final manuscript.

## Pre-publication history

The pre-publication history for this paper can be accessed here:

http://www.biomedcentral.com/1471-2393/12/23/prepub

## Supplementary Material

Additional file 1 **Description of the electronic search strategy used to perform the literature search**. Click here for file

Additional file 2 **List of excluded articles**. Click here for file

Additional file 3 **Meta-analyses performed with different variance estimators to generate pooled relative risks for the IADSPG and WHO criteria for gestational diabetes in the prediction of pregnancy outcomes**. Click here for file
