# Supplementary material for: Gestational diabetes and pregnancy outcomes - a systematic review of the World Health Organization (WHO) and the International Association of Diabetes in Pregnancy Study Groups (IADPSG) diagnostic criteria
Source: BMC Pregnancy Childbirth. 2012 Mar 31;12:23. doi: 10.1186/1471-2393-12-23 (PMC3352245; doi:10.1186/1471-2393-12-23)
Supplement: Additional file 1 — Description of the electronic search strategy used to perform the literature search. [file 1471-2393-12-23-S1.DOC]

**Additional File 1**

Electronic search of EMBASE for maternal outcomes.

| 1 | gestational diabetes.mp. or exp pregnancy diabetes mellitus/ |
| --- | --- |
| 2 | glucose intolerance.mp. or glucose intolerance/ |
| 3 | (glucose intolerance and pregnancy).mp. |
| 4 | 1 or 2 or 3 |
| 5 | cesarean section.mp. or exp cesarean section/ |
| 6 | "eclampsia and preeclampsia"/ or pregnancy toxemia/ |
| 7 | exp preeclampsia/ or exp "eclampsia and preeclampsia"/ or preeclampsia.mp. |
| 8 | pre-eclampsia.mp. |
| 9 | pregnancy induced hypertension.mp. or exp maternal hypertension/ |
| 10 | exp eclampsia/ or eclampsia.mp. |
| 11 | gestational hypertension.mp. |
| 12 | 6 or 7 or 8 or 9 or 10 or 11 |
| 13 | urinary tract infection.mp. or urinary tract infection/ |
| 14 | urinary infection.mp. |
| 15 | 13 or 14 |
| 16 | pregnancy/ |
| 17 | 15 and 16 |
| 18 | perineum injury/ or perineum laceration/ or perineum laceration.mp. |
| 19 | perineal laceration.mp. |
| 20 | perineal trauma.mp. |
| 21 | perineum trauma.mp. |
| 22 | 18 or 19 or 20 or 21 |
| 23 | 16 and 22 |
| 24 | delivery/ |
| 25 | obstetric*.mp. |
| 26 | 24 or 25 |
| 27 | 22 and 26 |
| 28 | 23 or 27 |
| 29 | instrumental delivery.mp. or instrumental delivery/ |
| 30 | obstetrical forceps.mp. or forceps/ |
| 31 | vacuum extraction.mp. or vacuum extraction/ |
| 32 | 26 and 31 |
| 33 | 29 or 30 or 32 |
| 34 | intensive care unit/ or intensive care/ |
| 35 | maternal intensive care.mp. |
| 36 | puerperium/ |
| 37 | 34 or 35 |
| 38 | puerperal.mp. |
| 39 | postpartum.mp. |
| 40 | 36 or 38 or 39 |
| 41 | 37 and 40 |
| 42 | maternal diabetes.mp. or maternal diabetes mellitus/ |
| 43 | 4 or 42 |
| 44 | 5 or 12 or 17 or 28 or 33 or 41 |
| 45 | 43 and 44 |
| 46 | case-control study.mp. or case control study/ |
| 47 | case control study.mp. |
| 48 | 46 or 47 |
| 49 | case report study.mp. |
| 50 | case-report study.mp. |
| 51 | 48 or 49 or 50 |
| 52 | 45 not 51 |

.mp. indicates a search of title, original title, abstract, name of substance word and subject heading word

Electronic search of PubMed for maternal outcomes.

| 1 | diabetes, gestational[MeSH Terms] |
| --- | --- |
| 2 | diabetes[All Fields] AND gestational[All Fields] |
| 3 | gestational diabetes[All Fields] |
| 4 | gestational[All Fields] AND diabetes[All Fields] |
| 5 | Diabetes, Gestational[Mesh] |
| 6 | glucose intolerance[MeSH Terms] |
| 7 | glucose[All Fields] AND intolerance[All Fields] |
| 8 | glucose intolerance[All Fields] |
| 9 | ((Glucose Intolerance[Mesh]) AND (pregnancy[MeSH Terms] OR pregnancy[All Fields])) |
| 10 | Search 1 OR 2 OR 3 OR 4 OR 5 OR 6 OR 7 OR 8 OR 9 |
| 11 | Cesarean Section[Mesh] |
| 12 | cesarean section[tw] |
| 13 | Pre-Eclampsia[Mesh] |
| 14 | Eclampsia[Mesh] |
| 15 | Hypertension, Pregnancy-Induced[Mesh] |
| 16 | preeclampsia[tw] |
| 17 | pre-eclampsia[tw] |
| 18 | eclampsia[tw] |
| 19 | gestational hypertension |
| 20 | pregnancy induced hypertension |
| 21 | Urinary Tract Infections[Mesh] AND Pregnancy[Mesh] |
| 22 | urinary infection AND pregnancy |
| 23 | perineum laceration OR perineal laceration AND delivery |
| 24 | Puerperal Infection[Mesh] |
| 25 | puerperal infection[tw] |
| 26 | Obstetrical Forceps[Mesh] OR instrumental delivery[tw] OR Vacuum Extraction, Obstetrical[Mesh] OR Extraction, Obstetrical[Mesh] |
| 27 | maternal admission ICU |
| 28 | maternal admission ITU |
| 29 | prolonged puerperal stay |
| 30 | prolonged post-partum stay |
| 31 | prolonged post partum stay |
| 32 | Search 11 OR 12 OR 13 OR 14 OR 15 OR 16 OR 17 OR 18 OR 19 OR 20 0R 21 0R 22 0R 23 0R 24 OR 25 OR 26 OR 27 OR 28 OR 29 OR 30 OR 31 |
| 33 | Case-Control Studies[Mesh] |
| 34 | case-control study[tw] |
| 35 | Case Reports [Publication Type] |
| 36 | Search 32 or 33 or 34 |

Electronic search of CINAHL for maternal outcomes.

| S1 | gestational diabetes |
| --- | --- |
| S2 | glucose intolerance |
| S3 | pregnancy |
| S4 | (pregnancy) and (S2 and S3) |
| S5 | S1 or S4 |
| S6 | Cesarean section |
| S7 | Pre-eclampsia |
| S8 | Eclampsia |
| S9 | pregnancy toxemia |
| S10 | pregnancy induced hypertension |
| S11 | gestational hypertension |
| S12 | S7 or S8 or S9 or S10 or S11 |
| S13 | Urinary tract infections |
| S14 | S3 and S13 |
| S15 | perineal laceration |
| S16 | perineum injury |
| S17 | perineum trauma |
| S18 | perineal trauma |
| S19 | S15 or S16 or S17 or S18 |
| S20 | (S15 or S16 or S17 or S18) and (S3) |
| S21 | delivery |
| S22 | (delivery) and (S19) |
| S23 | S20 or S22 |
| S24 | instrumental delivery |
| S25 | obstetrical forceps |
| S26 | vacuum extraction |
| S27 | (vacuum extraction) and (S21) |
| S28 | S24 or S25 or S27 |
| S29 | Intensive care unit |
| S30 | intensive care |
| S31 | S29 or S30 |
| S32 | Puerperium |
| S33 | Postpartum |
| S34 | S32 or S33 |
| S35 | S31 and S34 |
| S36 | or S12 or S14 or S20 or S28 or S35 |
| S37 | S6 or S12 or S14 or S20 or S28 or S35) and (S4) |

Similar search strategy was used to search for fetal outcomes on PubMed, EMBASE and CINAHL, using a combinations of the following keywords: fetal death, Stillbirth, macrosomia, Premature Birth, neonatal hypoglycemia, Hyperbilirubinemia, Neonatal, shoulder dystocia, Asphyxia Neonatorum, Respiratory Distress Syndrome, Newborn, prolonged neonatal stay

Electronic search of LILACS for maternal outcomes

| "gestational diabetes" or "glucose intolerance" AND "cesarean section" or (cesarean section) or "pre-eclampsia" or "eclampsia" or (pregnancy induced hypertension ) or (gestational hypertension) or (urinary tract infection pregnancy) or (perineal trauma) or ( perineum injury delivery) or (instrumental delivery) or (puerperal intensive care unit) |
| --- |

For the electronic search of IMEMR, IMSEAR, WHO-Afro library, IMSEAR, EMCAT, IMEMR, WPRIM and Cochrane Library (CENTRAL) we used a more broad search using a combination of terms “pregnancy” AND “diabetes” AND “gestational diabetes” for both, maternal and fetal outcomes.
