# Supplementary material for: Gestational diabetes and pregnancy outcomes - a systematic review of the World Health Organization (WHO) and the International Association of Diabetes in Pregnancy Study Groups (IADPSG) diagnostic criteria
Source: BMC Pregnancy Childbirth. 2012 Mar 31;12:23. doi: 10.1186/1471-2393-12-23 (PMC3352245; doi:10.1186/1471-2393-12-23)
Supplement: Additional file 2 — List of excluded articles. [file 1471-2393-12-23-S2.RTF]

Additional file 2 

 	J Gynecol Obstet 1995 1995; 48(3):331-339	
Abell DA, Beischer NA	Diabetes 1975; 24(10):874-880	
Abell DA, Beischer NA, Papas AJ, Willis MM	Am J Obstet Gynecol 1976; 124(4):388-392	
Abolfazl M, Hamidreza TS, Narges M, Maryam Y	JPMA 2008; 24(4):566-570	
Agardh CD, Aberg A, Norden NE	J Intern Med 1996; 240(5):303-309	
Agarwal MM, Dhatt GS, Punnose J, Koster G	Diabet Med 2005; 22(12):1731-1736	
Agarwal MM, Punnose J, Dhatt GS	Diabetes Res Clin Pract 2004; 63(1):73-74	
Aglan N	New Egypt J Med 1999; 20(5):303-309	
Al Mahroos S, Nagalla DS, Yousif W, Sanad H	Ann Saudi Med 2005; 25(2):129-133	
Al Mendalawi MD	Saudi Med J 2009; 30(4):583	
Alberico S, Strazzanti C, De Santo D, De Seta F, Lenardon P, Bernardon M, Zicari S, Guaschino S	J Matern Fetal Neonatal Med 2004; 16(6):331-337	
Al-Hakeem MM	J Fam Community Med 2006; 13(2):55-59	
Al-Najashi SS	Bahrain Med Bull 1997; 19(4):104-107	
Anderberg E, Kallen K, Berntorp K	Acta Obstet Gynecol Scand 2010; 89(12):1532-1537	
Athukorala C, Crowther CA, Willson K	Aust N Z J Obstet Gynaecol 2007; 47(1):37-41	
Bartha JL, Martinez-Del-Fresno P, Comino-Delgado R	Am J Obstet Gynecol 2000; 182(2):346-350	
Beischer NA, Wein P, Sheedy MT, Steffen B	Aust N Z J Obstet Gynaecol 1996; 36(3):239-247	
Berkus MD, Langer O	Obstet Gynecol 1993; 81(3):344-348	
Blaskova O, Sasko A, Razus M, Pohlova G, Chabda J, Veselska T, Kostolny I, Beckova A	Bratisl Lek Listy 1980; 73(2):136-142	
Bo S, Menato G, Gallo ML, Bardelli C, Lezo A, Signorile A, Gambino R, Cassader M, Massobrio M, Pagano G	Acta Obstet Gynecol Scand 2004; 83(4):335-340	
Bojadzhieva M, Atanassova I, Dimitrova V, Zacharieva S, Tankova T, Grozeva G, Todorova K, Stoikova V, Kedikova S	Akush Ginekol (Sofiia) 2010; 49(3):3-9	
Bonomo M, Corica D, Mion E, Goncalves D, Motta G, Merati R, Ragusa A, Morabito A	Diabet Med 2005; 22(11):1536-1541	
Botella LJ	An R Acad Nac Med (Madr) 1984; 101(2):153-184	
Bozhinova S, Slavov N, Pandurski F	Akush Ginekol (Sofiia) 1988; 27(3):33-37	
Breschi MC, Seghieri G, Bartolomei G, Gironi A, Baldi S, Ferrannini E	Diabetologia 1993; 36(12):1315-1321	
Bühling K, Stein U, Dudenhausen JW	Geburtsh Frauenheilk 1998; 58:100-109	
Campos MA, Reichelt AA, Facanha C, Forti AC, Schmidt MI	Braz J Med Biol Res 2008; 41(8):684-688	
Carr DB, Newton KM, Utzschneider KM, Faulenbach MV, Kahn SE, Easterling TR, Heckbert SR	Hypertens Pregnancy 2011; 30(2):153-163	
Chandna A, Zuberi LM, Munim S	Int J Gynaecol Obstet 2006; 94(2):119-120	
Chen X-M	Fudan University J of Med Sciences 2001; 28 (6):547-548	
Cheng YW, Block-Kurbisch I, Caughey AB	Obstet Gynecol 2009; 114(2 Pt 1):326-332	
Cheng YW, Esakoff TF, Block-Kurbisch I, Ustinov A, Shafer S, Caughey AB	J Matern Fetal Neonatal Med 2006; 19(11):729-734	
Cheng YW, McLaughlin GB, Esakoff TF, Block-Kurbisch I, Caughey AB	J Matern Fetal Neonatal Med 2007;20(12):903-908	
Chou CY, Lin CL, Yang CK, Yang WC, Lee FK, Tsai MS	J Womens Health (Larchmt ) 2010; 19(5):935-939	
Corrado F, Benedetto AD, Cannata ML, Cannizzaro D, Giordano D, Indorato G, Rizzo P, Stella NC, D'Anna R	J Matern Fetal Neonatal Med 2009; 22(7):597-601	
Cosson E, Benchimol M, Carbillon L, Pharisien I, Paries J, Valensi P, Lormeau B, Bolie S, Uzan M, Attali JR	Diabetes Metab 2006; 32(2):140-146	
Cousins L, Dattel B, Hollingsworth D, Hulbert D, Zettner A	Am J Obstet Gynecol 1985; 153(4):381-385	
Coustan DR	Diabetes Digest 2011; 7(5):5-15	
Coustan DR	Semin Perinatol 1994; 18(5):407-413	
Coustan DR, Carpenter MW	Clin Obstet Gynecol 1985; 28(3):507-515	
Crowther CA, Hiller JE, Moss JR, McPhee AJ, Jeffries WS, Robinson JS	N Engl J Med 2005; 352(24):2477-2486	
Curet LB, Izquierdo LA, Gilson GJ, Del Valle GO, Qualls C	J Matern Fetal Med 1997; 6(1):28-30	
de Sereday MS, Damiano MM, Gonzalez CD, Bennett PH	J Diabetes Complications 2003; 17(3):115-119	
Deerochanawong C, Putiyanun C, Wongsuryrat M, Serirat S, Jinayon P	Diabetologia 1996; 39(9):1070-1073	
Di Cianni G, Benzi L, Casadidio I, Orsini P, Rossi L, Fontana G, Malara N, Villani G, Di Carlo A, Trifiro R, Bottone P, Luchi C, Fantoni M, Teti G, Marselli L, Volpe L, Navalesi R	Ann Ist Super Sanita 1997; 33(3):389-391	
Diez JJ, Grande C, Pallardo LF, de la Morena ML, Ibars MT	Med Clin (Barc ) 1989; 93(2):41-45	
Elkind-Hirsch KE, Ogden BW, Darensbourg CJ, Schelin CL	IJDM 2010; 2(1):3-5	
Ertunc D, Tok E, Dilek U, Pata O, Dilek S	Ann Saudi Med 2004; 24(4):280-283	
Fadl HE, Ostlund IK, Magnuson AF, Hanson US	Diabet Med 2010; 27(4):436-441	
Forest JC, Masse J, Garrido-Russo M	Clin Biochem 1994; 27(4):299-304	
Forsbach G, Contreras-Soto JJ, Fong G, Flores G, Moreno O	Diabetes Care 1988; 11(3):235-238	
Fraser R	Br J Obstet Gynaecol 1995; 102(4):275-277	
Garshasbi A, Zamiry A, Faghihzadeh S, Naghizadeh MM	J Zanjan Univ Med Sci Health Serv 2010; 18 (71):1-12	
Giampietro O, Matteucci E	Ann Ist Super Sanita 1997; 33(3):399-402	
Godwin M, Muirhead M, Huynh J, Helt B, Grimmer J	CMAJ 1999; 160(9):1299-1302	
Gokcel A, Bagis T, Killicadag EB, Tarim E, Guvener N	J Endocrinol Invest 2002;25(4):357-361	
Gorgojo Martinez JJ, Almodovar RF, Lopez HE, Donnay CS	Revista Clinica Espanola 2002;(3):136-141	
Greco P, Loverro G, Selvaggi L	Gynecol Obstet Inves 1994; 37(4):242-245	
Greene MF	N Engl J Med 1997 1997;337(22):1625-1626	
Griffin ME, Coffey M, Johnson H, Scanlon P, Foley M, Stronge J, O'Meara NM, Firth RG	Diabet Med 2000;17(1):26-32	
Gumus II, Turhan NO	J Obstet Gynaecol Res 2008;34(3):359-363	
Hadden DR	Diabetes Care 1980; 3(3):440-446	
Hawthorne G, Robson S, Ryall EA, Sen D, Roberts SH, Ward Platt MP	BMJ 1997;315(7103):279-281	
Hedderson MM, Ferrara A, Sacks DA	Obstetrics and Gynecology 2003; 102(4):850-856	
Herman G, Raimondi B	Am J Perinat 1988;5(2):168-171	
Hillier TA, Vesco KK, Pedula KL, Beil TL, Whitlock EP, Pettitt DJ	Ann Intern Med 2008; 148(10):766-775	
Hod M, Merlob P	Early Pregnancy 1996; 2(1):15-17	
Hoet JJ	Diabetes Care 1980; 3(3):497-498	
Hopp H, Vollert W, Ebert A, Weitzel H, Glockner E, Jahrig D	Geburtshilfe Frauenheilkd 1995; 55(5):275-279	
Hossein-Nezhad A, Maghbooli Z, Vassigh A-R, Larijani B	Taiwan J Obstet Gynecol 2007; 46(3):236-241	
Hughes PF, Agarwal M, Newman P, Morrison J	Diabetes Res Clin Pract 1995;28(1):73-78	
Hughes PF, Agarwal M, Thomas L	J Obstet Gynaecol 1997;17(6):540-544	
Huidobro MA, Fulford A, Carrasco PE	Rev Med Chile 2004;132(8):931-938	
Jacobson JD, Cousins L	Am J Obstet Gynecol 1989;161(4):981-986	
James WH	Diabetes Care 2001;24(11):2018-2019	
Jang HC	J Korean Diabetes Assoc 2002; 26(4):221-228	
Jang HC, Cho YM, Park KS, Kim SY, Lee HK, Kim MY, Yang JH, Shin SM	J Korean Diabetes Assoc 2004; 28(2):122-130	
Jensen DM, Damm P, Sorensen B, Molsted-Pedersen L, Westergaard JG, Klebe J, Beck-Nielsen H	Am J Obstet Gynecol 2001;185(2):413-419	
Jensen DM, Damm P, Sorensen B, Molsted-Pedersen L, Westergaard JG, Korsholm L, Ovesen P, Beck-Nielsen H	Diabet Med 2003; 20(1):51-57	
Jensen DM, Damm P, Sorensen B, Molsted-Pedersen L, Westergaard JG, Ovesen P, Beck-Nielsen H	Am J Obstet Gynecol 2003; 189(1):239-244	
Jensen DM, Korsholm L, Ovesen P, Beck-Nielsen H, Molsted-Pedersen L, Damm P	Acta Obstet Gynecol Scand 2008; 87(1):59-62	
Joseph SE	Diabet Med 1996;13(12):1072	
Ju H, Rumbold AR, Willson KJ, Crowther CA	BMC pregnancy and childbirth 2008; 8:31	
Kaufmann RC, McBride P, Amankwah KS, Huffman DG	Obstetrics and Gynecology 1992; 80(1):97-101	
Kautzky-Willer A, Bancher-Todesca D, Weitgasser R, Prikoszovich T, Steiner H, Shnawa N, Schernthaner G, Birnbacher R, Schneider B, Marth C, Roden M, Lechleitner M	J Clin Endocrinol Metab 2008; 93(5):1689-1695	
Keshavarz M, Cheung NW, Babaee GR, Moghadam HK, Ajami ME, Shariati M	Diabetes Res Clin Pract 2005; 69(3):279-286	
Khan KS, Daya S	Int J Gynaecol Obstet 1996; 53(2):111-116	
Khan KS, Hashmi FA, Rizvi JH	J Pak Med Assoc 1995; 45(7):176-179	
Koh KS	Korean J Perinatol 2000; 11(4): 421-429	
Korucuoglu U, Biri A, Turkyilmaz E, Doga YF, Ilhan M, Hirfanoglu IM, Atalay Y	Diabetes Res Clin Pract 2008; 80(1):69-74	
Kvetny J, Poulsen HF, Damgaard DW	Danish Medical Bulletin 1999; 46(1):57-59	
Kwik M, Seeho SKM, Smith C, McElduff A, Morris JM	Diabetes Res Clin Pract 2007; 77(2):263-268	
Landon MB	Am J Obstet Gynecol 2010; 202(6):649-653	
Landy HJ, Gomez-Marin O, O'Sullivan MJ	Obstetrics and Gynecology 1996; 87(3): 395-400	
Langer O, Anyaegbunam A, Brustman L, Divon M	Am J Obstet Gynecol 1989; 161(3):593-599	
Langer O, Brustman L, Anyaegbunam A, Mazze R	Am J Obstet Gynecol 1987; 157(3):758-763	
Langer O, Maulik D	J Matern Fetal Neonatal Med 2002; 11(4):217	
Lao TT, Ho LF	J Soc Gynecol Investig 2003; 10(6):366-371	
Lapolla A, Bonomo M, Dalfra MG, Parretti E, Mannino D, Mello G, Di Cianni G	Diabetes Metab 2010; 36(4):265-270	
Lapolla A, Dalfra MG, Bonomo M, Castiglioni MT, Di Cianni G, Masin M, Mion E, Paleari R, Schievano C, Songini M, Tocco G, Volpe L, Mosca A	Diabetes Res Clin Pract 2007; 77(3):465-470	
Lauszus FF, Paludan J, Klebe JG	Acta Obstet Gynecol Scand 1999; 78(6): 520-525	
Ledyard R	Am Fam Physician 1997; 55(5): 1583-1584	
Lepercq J	Gynecologie Obstetrique Fertilite 2006; 34(7-8):571-57	
Lepercq J	Gynecologie Obstetrique Fertilite 2007; 35(10):1080	
LI Shuang-di;ZHANG Jia-rong;XIE Yi;et al. 	Chin J Perinat Med 2007. (04)	
Lin C-H, Wen S-F, Wu Y-H, Huang M-J	Chang Gung Medical Journal 2009; 32(3):283-289	
Lindsay MK, Graves W, Klein L	Obstetrics and Gynecology 1989;73(1):103-106	
Lindsay RS	Diabetes 2009;58(2):302-303	
Little RR, McKenzie EM, Shyken JM, Winkelmann SE, Ramsey LM, Madsen RW, Goldstein DE	Diabetes Care 1990; 13(5):483-487	
Lopez-de la Pena XA, Cajero Avelar JJ, Leon Romo LF	Arch Med Res 1997; 28(2):281-284	
Lu YP, Sun GS, Weng XY, Mao L, Li LA	Zhonghua Fu Chan Ke Za Zhi 2003; 38(12):729-732	
Lurie S, Levy R, Weiss R, Boultin G, Hagay ZJ	J Obstet Gynaecol 1998;28(5):451-454	
Marini M, Chabot VA, Stamm H, Berger W	Schweiz Med Wochenschr Suppl 1994; 60:47-52	
Mello G, Parretti E, Cioni R, Lucchetti R, Carignani L, Martini E, Mecacci F, Lagazio C, Pratesi M	Diabetes Care 2003; 26(4):1206-1210	
Mello G, Parretti E, Mecacci F, Lucchetti R, Lagazio C, Pratesi M, Scarselli G	Eur J Endocrinol 1997;137(1):27-33	
Moses RG, Calvert D	Diabetes Care 1995;18(12):1527-1533	
Moses RG, Griffiths RD	J Soc Gynecol Investig 1995; 2(3):523-525	
Mustafa, F.E.; Sandhu, A.K.	East Mediterr Health J 1998; 4 (3): 585-588	
Nasrat H, Fageeh W, Abalkhail B, Yamani T, Ardawi MSM	Int J Gynaecol Obstet 1996;53(2):117-123	
Nasrat HA, Ardawi MS, Abalkhail BA	Diabet Med 1996;13(10):861-867	
Naylor CD, Sermer M, Chen E, Sykora K	J Amer Medi Assoc 1996; 275(15):1165-1170	
Nord E, Hanson U, Persson B	Acta Obstet Gynecol Scand 1995;74(8):589-593	
Nuttens MC, Fournie A	Diabetes Metab 1997;23:9-14	
Olarinoye JK, Ohwovoriole AE, Ajayi GO	West Afr J Med 2004;23(3):198-201	
Olofsson P, Liedholm H, Sartor G, Sjoberg NO, Svenningsen NW, Ursing D	Acta Obstet Gynecol Scand Suppl 1984; 122:3-62:3-62	
Ostlund I, Hanson U, Bjorklund A, Hjertberg R, Eva N, Nordlander E, Swahn ML, Wager J	Diabetes Care 2003; 26(7):2107-2111	
O'Sullivan JB	Adv Metab Disord 1970; 1:Suppl	
O'Sullivan JB, Charles D, Mahan CM, Dandrow RV	Am J Obstet Gynecol 1973; 116(7):901-904	
Pacora Portella P	Diagnostico (Peru) 1995; 34(5):15-21	
Pacora Portella P, Huiza Espinoza L, Santiva+¦ez Pimentel AC, Buzzio Veramendi Y, Ayala Arias M+	Rev Soc Peru Med Interna 2002; Array 190-195	
Pehrson SL	Acta Obstet Gynecol Scand Suppl 1974; 33:1-152	
Pettitt DJ	Diabetes care 2001; 24(7):1129	
Pettitt DJ, Bennett PH, Hanson RL, Narayan KMV, Knowler WC	Diabetes Care 1994; 17(11):1264-1268	
Pettitt DJ, Knowler WC, Baird HR, Bennett PH	Diabetes Care 1980; 3(3):458-464	
Philipson EH, Kalhan SC, Rosen MG, Edelberg SC, Williams TG, Riha MM	Diabetes 1985; 34 Suppl 2:55-60	
Poncet B, Touzet S, Rocher L, Berland M, Orgiazzi J, Colin C	Eur J Obstet Gynecol Reprod Biol 2002; 103(2):122-129	
Pribylova H, Dvorakova L	Acta Diabetol 1996;33(1):30-34	
Ramachandran A, Snehalatha C, Clementina M, Sasikala R, Vijay V	Diabetes Res Clin Pract 1998;41(3):185-189	
Ramirez Torres MA	Ginecol Obstet Mex 2005;73(9):484-491	
Ramtoola S, Home P, Damry H, Husnoo A, Ah-Kion S	BMJ 2001;322(7293):1025-1026	
Retnakaran R, Qi Y, Sermer M, Connelly PW, Zinman B, Hanley AJ	Diabetes care 2008;31(7):1275-1281	
Rey E, Monier D, Lemonnier M-C	Clin Invest Med 1996;19(6):406-415	
Ricart W, Lopez J, Mozas J, Pericot A, Sancho MA, Gonzalez N, Balsells M, Luna R, Cortazar A, Navarro P, Ramirez O, Flandez B, Pallardo LF, Hernandez A, Ampudia J, Fernandez-Real JM, Corcoy R	Diabetologia 2005;48(6):1135-1141	
Ricart W, Lopez J, Mozas J, Pericot A, Sancho MA, Gonzalez N, Balsells M, Luna R, Cortazar A, Navarro P, Ramirez O, Flandez B, Pallardo LF, Hernandez A, Ampudia J, Fernandez-Real JM, Hernandez-Aguado I, Corcoy R	J Epidemiol Commun H  2009; 63(1):64-68	
Riskin-Mashiah S, Younes G, Damti A, Auslender R	Diabetes Care 2009;32(9):1639-1643	
Rizvi JH, Rasul S, Malik S, Rehamatuallh A, Khan MA	Asia Oceania J Obstet Gynaecol 1992; 18(2):99-105	
Ros Wendland EM, Duncan BB, Belizan JM, Vigo A, Schmidt MI	Arq Bras Endocrinol Metab 2008;52(6):975-984	
Rudge MV, Calderon IM, Ramos MD, Abbade JF, Rugolo LM	Gynecol Obstet Invest 2000; 50(2):108-112	
Sacks DA, Greenspoon JS, Abu-Fadil S, Henry HM, Wolde-Tsadik G, Yao JFF	Am J Obstet Gynecol 1995;172(2 I):607-614	
Saldana TM, Siega-Riz AM, Adair LS, Savitz DA, Thorp JM, Jr	Diabetes Care 2003; 26(3):656-661	
Samad N, Hassan JA, Shera AS, Maqsood A	JPMA 1996;46(11):249-252	
Savona-Ventura C, Chircop M	J Diabetes Complications 2008; 22(3):178-180	
Savona-Ventura C, Craus J, Vella K, Grima S	Malta Medical Journal 2010;22(1):18-20	
Sayeed MA,  	Diabet Med 2005; 22(9):1267-1271	
Schmitz T	Gynecol Obstet Fertil 2008; 36(5):567-569	
Scholl TO, Sowers M, Chen X, Lenders C	Am J Epidemiol 2001;154(6):514-520	
Schrader HM, Jovanovic-Peterson L, Bevier WC, Peterson CM	Am J Perinat 1995;12(4):247-251	
Scott DA, Loveman E, McIntyre L, Waugh N	Health Technology Assessment 2002;6(11)	
Seghieri G, Breschi MC, Innocenti C, Anichini R, De Giorgio LA, Ferrannini E	Diabetes Nutr Metabol Clin Exp 1996; 9(3):139-144	
Sepe SJ, Connell FA, Geiss LS, Teutsch SM	Diabetes 1985; 34 Suppl 2:13-6:13-16	
Serirat S, Deerochanawong C, Sunthornthepvarakul T, Jinayon P	J Med Assoc Thai 1992; 75(6):315-319	
Sermer M, Naylor CD, Farine D, Kenshole AB, Ritchie JWK, Gare DJ, Cohen HR, McArthur K, Holzapfel S, Biringer A	Diabetes Care 1998; 21(SUPPL2):B33-B42	
Sermer M, Naylor CD, Gare DJ, Kenshole AB, Ritchie JWK, Farine D, Cohen HR, McArthur K, Holzapfel S, Biringer A, Chen E	Am J Obstet Gynecol 1995;173(1):146-156	
Shaukat A, Arain TM, Abid S, Mahmud R	J Coll Physicians Surg Pak 1999;9(5):211-214	
Siribaddana SH, Deshabandu R, Rajapakse D, Silva K, Fernando DJ	Ceylon Med J 1998; 43(2):88-91	
Skyler JS, O'Sullivan MJ, Robertson EG, Skyler DL, Holsinger KK, Lasky IA, McLeod AG, Burkett G, Mintz DH	Diabetes Care 1980; 3(1):69-76	
Soonthornpun S, Soonthornpun K, Aksonteing J, Thamprasit A	Diabetes Res Clin Pract 2009; 85(2):203-207	
Stone CA, McLachlan KA, Halliday JL, Wein P, Tippett C	Med J Aust 2002;177(9):486-491	
Sun B, Wang X, Song Q, Wang Y, Xue L, Wang C, Quan Z, Zhang Y, Niu P	Chin Med J (Engl) 1995; 108(12):910-913	
Sun BZ, Wang XG, Wang YL	Zhonghua fu chan ke za zhi 1994 189;29(3):141-143	
Svare JA, Hansen BB, Molsted-Pedersen L	Acta Obstet Gynecol Scand 2001; 80(10):899-904	
Tallarigo L, Giampietro O, Penno G, Miccoli R, Gregori G, Navalesi R	N Engl J Med 1986; 315(16):989-992	
Tamez Perez HE, Rodriguez AM, Trevino HM, Espinosa CJ, Salas Galindo LR, Barquet BJ, Paez Jimenez FJ	Rev Invest Clin 1993;45(5):453-456	
Tan PC, Ling LP, Omar SZ	Int J Gynaecol Obstet  2009;105(1):50-55	
Tardioli MC, Massi BM, Angeli G, Damiani F, Frascarelli A, Gigliarelli D, Masci D, Narducci P, Norgiolini R, Nicolucci A, Pastorelli G, Rossi AL, Settonce S, Toppetti ML, Brunetti P	Diabetes Nutr Metabol Clin Exp 1993; 6(6):377-380	
Thanasuan S, Borriboonhirunsarn D	J Med Assoc Thai 2006;89(8):1109-1114	
Thomas A, Kaur S, Somville T	Saudi Med J 2002;23(7):814-818	
Tieu J, Crowther CA, Middleton P, McPhee AJ	Cochrane Database Syst Rev 2008 Article Number: CD007222 	
Truscello AM, Hollingsworth DR, Felice ME, Shragg P	J Adolesc Health Care 1988; 9(2):150-155	
Verma A, Mitchell BF, Demianczuk N, Flowerdew G, Okun NB	J Matern Fetal Med 1997; 6(3):187-193	
Vignoles P, Gire C, Mancini J, Bretelle F, Boubli L, Janky E, Carcopino X	Arch Gynecol Obstet 2010;284(5): 1099-104	
Voldner N, Froslie KF, Bo K, Haakstad L, Hoff C, Godang K, Bollerslev J, Henriksen T	Acta Obstet Gynecol Scand 2008; 87(4):423-429	
Voldner N, Qvigstad E, Froslie KF, Godang K, Henriksen T, Bollerslev J	J Matern Fetal Neonatal Med 2010; 23(1):74-81	
Walters BNJ	Aust N Z J Obstet Gynaecol  2006; 46(5):463-464	
Weeks JW, Major CA, De Veciana M, Morgan MA	Am J Obstet Gynecol 1994;171(4):1003-1007	
Weijers RNM, Bekedam DJ, Smulders YM	Diabetes Care 2002;25(1):72-77	
Weiner CP	Am J Obstet Gynecol 1988;159(4):862-870	
Weiss BD, Senf JH, Udall, O'Connor PJ	J Fam Practice 1989;29(4):389-396	
Wen SW, Liu S, Kramer MS, Joseph KS, Levitt C, Marcoux S, Liston RM	Am J Epidemiol 2000; 152(11):1009-1014	
Wu QK, Luo LM, Li P, Gu JH, Feng J	Int J Gynaecol Obstet 2005; 88(2):122-126	
Yang X, Hsu-Hage B, Zhang H, Zhang Y, Zhang C	Diabetes Care 2002;25(9):1619-1624	
Yang X, Zhang H, Dong L, Yu S, Guo Z, Hsu-Hage BH	J Diabetes Complications 2004; 18(1):37-41	
Yogev Y, Langer O, Xenakis EMJ, Rosenn B	J Matern Fet Neonatal Med 2005;17(1):29-34	
