# Supplementary material for: Gestational diabetes and pregnancy outcomes - a systematic review of the World Health Organization (WHO) and the International Association of Diabetes in Pregnancy Study Groups (IADPSG) diagnostic criteria
Source: BMC Pregnancy Childbirth. 2012 Mar 31;12:23. doi: 10.1186/1471-2393-12-23 (PMC3352245; doi:10.1186/1471-2393-12-23)
Supplement: Additional file 3 — Meta-analyses performed with different variance estimators to generate pooled relative risks for the IADSPG and WHO criteria for gestational diabetes in the prediction of pregnancy outcomes. [file 1471-2393-12-23-S3.DOC]

Additional file 3

|  | REML | | ML | | Empirical Bayes | | Sidik-Jonkman | | DerSimonean and Laird | | Fixed effects model | |
| --- | --- | --- | --- | --- | --- | --- | --- | --- | --- | --- | --- | --- |
|  | RR | I^2^ | RR | I^2^ | RR | I^2^ | RR | I^2^ | RR | I^2^ | RR | I^2^ |
| WHO |  |  |  |  |  |  |  |  |  |  |  |  |
| Perinatal Mortality | 1.55 (0.88-2.73) | 0 | 1.55 (0.88-2.73) | 0 | 1.55 (0.88-2.73) | 0 | 1.57 (0.83-2.97) | 5 | 1.55 (0.88-2.73) | 0 | 1.55 (0.88-2.73) | - |
| Macrosomia | 1.81 (1.47-2.22) | 0 | 1.81 (1.47-2.22) | 0 | 1.81 (1.47-2.22) | 0 | 1.90 (1.40-2.59) | 29 | 1.81 (1.47-2.22) | 0 | 1.81 (1.47-2.22) | - |
| LGA births | 1.53 (1.39-1.69) | 0 | 1.53 (1.39-1.69) | 0 | 1.53 (1.39-1.69) | 0 | 1.53 (1.38-1.70) | 1 | 1.53 (1.39-1.69) | 0 | 1.53 (1.39-1.69) | - |
| Preeclampsia | 1.69 (1.31-2.18) | 38 | 1.61 (1.40-1.85) | 0 | 1.70 (1.30-2.23) | 43 | 1.72 (1.27-2.32) | 52 | 1.70 (1.31-2.20) | 40 | 1.61 (1.40-1.85) | - |
| Caesarean delivery | 1.37 (1.24-1.51) | 29 | 1.38 (1.29-1.47) | 0 | 1.37 (1.27-1.49) | 13 | 1.37 (1.20-1.57) | 51 | 1.37 (1.26-1.49) | 18 | 1.38 (1.29-1.47) | - |
| IADPSG |  |  |  |  |  |  |  |  |  |  |  |  |
| LGA births | 1.73 (1.28-2.35) | 93 | 1.75 (1.37-2.22) | 89 | 1.73 (1.27-2.37) | 93 | 1.73 (1.27-2.36) | 93 | 1.75 (1.39–2.20) | 87 | 1.90 (1.76-2.03) | - |
| Preeclampsia | 1.71 (1.38-2.13) | 73 | 1.73 (1.46-2.06) | 58 | 1.72 (1.40-2.11) | 70 | 1.72 (1.40-2.11) | 70 | 1.71 (1.37-2.14) | 74 | 1.81 (1.64-1.99) | - |
| Caesarean delivery | 1.23 (1.01-1.51) | 93 | 1.23 (1.04-1.46) | 90 | 1.23 (1.01-1.51) | 93 | 1.23 (1.01-1.50) | 93 | 1.23 (0.99-1.53) | 94 | 1.28 (1.22-1.35) | - |

REML – Restricted; Maximum Likelihood; ML – Maximum Likelihood; I^2^ - Inconsistency indexes in %
